# Regulatory challenges of digital health: the case of mental health applications and personal data in South Africa

**DOI:** 10.3389/fphar.2025.1498600

**Published:** 2025-04-30

**Authors:** Marietjie Botes

**Affiliations:** School of Law, University of KwaZulu Natal, Durban, South Africa

**Keywords:** digital mental health, data protection, privacy regulation, AI-driven mental health tools, South African legal framework, mental health data security

## Abstract

**Introduction:**

This study explores the regulatory challenges posed by digital mental health applications in South Africa, particularly regarding the collection and protection of personal data. It aimed to assess whether South Africa’s current legal framework sufficiently protects users’ sensitive mental health data amidst the rise of digital mental health solutions, especially in the context of privacy concerns.

**Methods:**

The research focused on the intersection of digital mental health applications, data protection laws, and user privacy in South Africa. It examined existing legal frameworks, including the Protection of Personal Information Act (POPIA), National Health Act (NHA), and Consumer Protection Act (CPA). The study reviewed relevant literature, legal texts, and case studies, focusing on mental health applications in both urban and rural contexts.

**Results:**

While South Africa has laws in place to protect personal information, these laws have significant gaps in addressing the unique risks associated with digital mental health technologies. Key findings include inadequate regulation of AI-driven mental health tools, insufficient guidelines for third-party data sharing, and challenges with cross-border data transfers.

**Discussion:**

The implications of these findings suggest that South Africa needs to modernize its legal framework to better regulate digital mental health tools and ensure user privacy. This includes improving AI regulation, strengthening consent mechanisms, and enhancing protections against third-party data misuse. Future research should focus on developing specific legal guidelines for mental health data and addressing the vulnerabilities faced by rural populations with low digital literacy. The study’s conclusions align with global concerns over the ethical implications of mental health datacommodification and emphasize the need for robust, adaptable regulatory approaches.

## 1 Introduction

Mental health applications, such as wellness trackers, teletherapy platforms, and self-help tools, have gained significant traction in South Africa, particularly in response to the COVID-19 pandemic (Goldschmidt et al., 2021). Apps like Mindful Revolution and Wysa became go-to resources, offering a variety of services from self-care routines to virtual consultations (Goodings et al., 2024). These digital solutions have provided much-needed accessibility to mental health support in a country where mental healthcare services are often inadequate due to systemic constraints and socio-economic disparities. However, the rapid growth of digital mental health technologies has outpaced regulatory oversight, exposing users to risks related to data privacy, security, and ethical concerns (Martinez-Martin and Kreitmair, 2018). Despite recognising the intersection of digital health technologies and data privacy, existing South African legal frameworks fail to adequately address the specific regulatory hurdles posed by mental health applications, especially in a country where digital literacy vary, and inequalities persist (Mishi and Anakpo, 2022). While laws such as the Protection of Personal Information Act (POPIA), the National Health Act (NHA), and the Consumer Protection Act (CPA) offer general guidelines on data protection, they do not provide sufficient clarity on how mental health data, particularly those collected through AI-driven applications, should be handled. Many digital mental health applications incorporate AI for diagnostics, chatbots, and behavioural assessments, yet South African regulations do not specify how AI-generated mental health insights should be validated or regulated to prevent harm and misinformation. Mental health applications also collect highly sensitive personal data, but current laws do not impose stringent security standards specific to digital mental health tools. The lack of guidance on data deidentification, encryption requirements, and third-party data-sharing practices leaves users vulnerable to data breaches and exploitation. In addition, many mental health applications operate on international cloud-based platforms, raising concerns about how South African users’ data is stored, processed, and shared across jurisdictions. While POPIA restricts data transfers to countries with equivalent protections, it does not outline specific mechanisms for compliance or enforcement, leaving significant regulatory uncertainty. The absence of a regulatory framework distinguishing clinically validated applications from non-evidence-based tools allows potentially harmful or misleading applications to proliferate without oversight. Unlike medical devices regulated under the Medicines and Related Substances Act, general mental health apps that do not provide direct clinical interventions often evade regulatory scrutiny. The implications of these challenges are particularly concerning given that mental health data is deeply personal and, if misused, could lead to stigma, discrimination, and psychological harm. Ensuring user trust in digital mental health tools require clear, enforceable regulations that balance innovation with ethical and legal protections. Moreover, regulatory interventions should not only constrain harmful practices but also foster the improvement of digital mental health technologies through mechanisms such as independent audits, AI transparency requirements, and ethical AI guidelines for mental health applications.

## 2 The rise of digital mental health applications

The digital mental health landscape in South Africa has seen the emergence of a diverse range of applications, each serving different aspects of mental healthcare. These apps can be broadly categorized into the following types: 1) Teletherapy Platforms: Apps such as BetterHelp and MyTherapist provide virtual therapy sessions with licensed mental health professionals and gained popularity during COVID-19 when in-person therapy was difficult (Jo et al., 2023); 2) Mindfulness and Meditation Apps: Applications like Headspace, Calm, and Insight Timer offer guided meditation, mindfulness exercises, and stress reduction techniques which are widely used in South Africa to promote relaxation and emotional well-being (Li and Leshed, 2022); 3) Self-Help and Cognitive Behavioral Therapy (CBT): Apps like Wysa and Moodpath focus on cognitive behavioral techniques, helping users identify and manage negative thoughts and behaviors that often include chatbots or pre-programmed exercises designed to reduce symptoms of anxiety, depression, and stress (Pavlopoulos et al., 2024); 4) Mental Health Monitoring Tools: Some apps, such as Sanvello and Youper, allow users to track their moods and mental health symptoms over time, offering insights into patterns and triggers for emotional states which encourage proactive self-care and provide resources for managing mental health (Jain et al., 2024); and 5) Peer Support Networks: Apps like 7 Cups create digital communities where individuals can connect with others experiencing similar challenges, promoting a sense of 2 belonging and support (Parkinson et al., 2022).

The uptake of mental health apps in South Africa has been driven by the accessibility of smartphones, the growing awareness of mental health issues, and the demand for cost-effective mental health support. The availability of both free and subscription-based models has expanded the user base, allowing individuals from different socio-economic backgrounds to access mental health resources. South Africa’s digital mental health app users often include university and school-aged students that are particularly drawn to self-help and mindfulness apps for managing academic pressures and stress (Gbollie et al., 2023). These apps are also useful to individuals juggling workplace stress and personal well-being, often in conjunction with in-person therapy as a supplement to their treatment plans (Sharma-Misra et al., 2023).

Several studies support the effectiveness of digital mental health applications in reducing symptoms of anxiety, depression, and stress (Kim et al., 2023). However, while an Australian study that evaluated MoodGym, a CBT-based app, found that users reported a significant reduction in symptoms of depression compared to a control group that received no intervention (Twomey and O’Reilly, 2017), the demographic, digital accessibility, and digital literacy in a LMIC country such as South Africa differ significantly from that of a HIC like Australia. In this context, Mindu et al. explored the potential and obstacles associated with deploying digital mental health solutions for young people in rural South Africa, specifically focusing on the Ingwavuma area in KwaZulu-Natal (Mindu et al., 2023). They found that mental health literacy in general was notably low in the study area, with only 22% of participants having received prior mental health education. Despite most participants (91%) having access to smartphones and the internet (87%), awareness and use of digital mental health apps were very low. Only around 50% of participants had heard of mental health apps, but none had used them. In contrast, MoodGYM, the widely available CBT app studied by Twomey et al., has been well studied and implemented across several regions in Australia. Key barriers identified in the South African context include the high cost of data with many participants struggled to afford the data required to use digital platforms; concerns over privacy and confidentiality were prevalent, especially when using social media; some participants mentioned religious or cultural beliefs that discouraged the use of digital platforms; many participants expressed that digital apps needed to be simpler to accommodate users with lower digital literacy; and a lack of apps in native languages limited access (Mindu et al., 2023). These factors hinder the uptake of mental health apps despite widespread smartphone ownership and clearly show how the barriers in HIC like Australia differ from those in rural South Africa, where infrastructure and socio-economic factors play a more significant role. For these interventions to be successful in South Africa, they need to be tailored to the local context. This includes addressing data costs, ensuring privacy and confidentiality, and making apps more user-friendly and culturally relevant.

In addition, despite the promise of digital mental health apps, a significant challenge also lies in the proliferation of non-evidence-based applications. Many apps available on app stores claim to provide mental health benefits but lack clinical validation or oversight by qualified mental health professionals (Gordon et al., 2020). These apps can potentially offer misleading advice, inadequate support, or even harm users by trivializing serious mental health conditions. The lack of regulation around mental health apps, including in South Africa, exacerbates this issue, as there are no clear guidelines for evaluating the safety and efficacy of these tools (Gooding, 2019). This leaves users vulnerable to the risks posed by apps that may prioritize engagement and profitability over evidence-based care.

## 3 Data collection and privacy concerns

The collection and use of data by mental health apps and neurotechnology products raises serious privacy and ethical concerns. While mental health apps like Calm and Headspace mainly collect behavioral and interaction data, consumer neurotechnology taps into deeper, more intimate neural data that can reveal thoughts and mental states. As neurotechnology becomes more widespread, it is crucial to address the ethical implications of mental privacy, data commodification, and the need for strong regulations to protect individuals’ brainwave data from misuse.

In addition to personal information such as a user’s name, email, payment details, and profile information, mental health apps can also collect usage data such as the frequency of app usage, interactions within the app, and time spent on specific activities like meditation sessions; device information such as IP addresses, device identifiers, browser type, and operating system; a user’s approximate location based on IP addresses; information related to the user’s mental health and well- being, often through questionnaires or tracking logs of feelings and emotional states; and a user’s personalized experience which may improving app performance and be used for marketing purposes; even data regarding users’ mental health status, therapy history, and session notes shared with therapists (Mendes et al., 2022). In addition to mental health apps, consumer neurotechnology products, such as brainwave activity trackers (e.g., Muse, NeuroSky), and brain-computer interfaces (BCIs), have emerged as popular tools for improving mental focus, meditation, and cognitive performance (Fontanillo Lopez et al., 2020). These products collect sensitive neural data to offer insights into brain activity and mental states. Most neurotechnology products collect electroencephalogram (EEG) data, which records electrical activity in the brain and is used to monitor different states such as relaxation, focus, or sleep (Soufineyestani et al., 2020). Some products may attempt to assess attention span, cognitive load, or mood based on brainwave patterns, while products like Emotiv and Muse may analyze EEG data to infer emotional states, such as stress levels or calmness (Baig and Kavakli, 2019). These products often combine brainwave data with other biometric data such as heart rate or galvanic skin response to provide a more comprehensive profile of the user’s mental and emotional state (Kaklauskas et al., 2022).

Neural data is highly sensitive as it can reflect mental health states, cognitive abilities, and emotional well-being which raises concerns about how this data is stored, used, and shared with third parties (Khalsa et al., 2018). The neural data collected may enable companies to make inferences about a person’s personality, behavior, or even predict future mental health issues which in turn raises ethical questions about the exploitation of such deeply personal information (Bzdok and Yeo, 2017). Brainwave data could also, in theory, be used for surveillance or manipulation, especially in contexts where neurotechnology becomes integrated with workplace or educational settings (Farahany, 2023). Lastly, neurodata could be commodified, leading to potential abuse by third parties, such as insurance companies or employers, to assess mental health risks or cognitive abilities, thus creating new forms of discrimination (Bublitz, 2022).

The commodification of neural data refers to the process of turning this sensitive information into a marketable product. In this way companies developing neurotechnology may monetize users’ brainwave activity data by selling insights to advertisers, insurance companies, or other third parties, like the way personal data from social media platforms is currently commodified (Vogel, 2023). The commercialization of neural data can lead to significant privacy violations, as companies might exploit this data for profit, undermining users’ autonomy and confidentiality (George, 2024). Users may further unknowingly surrender highly valuable and intimate data for minimal or no compensation, with companies reaping economic benefits from selling this data to third parties (Hoffman, 2022). Commodifying neural data without adequate regulation could worsen societal inequalities. For example, people with diagnosed mental health conditions could face discrimination in employment or healthcare if their neural data is accessed by organizations making biased decisions (Timmons et al., 2023).

While mental health applications offer accessible and scalable solutions for mental healthcare, their data collection practices raise significant privacy and ethical concerns. Many of these apps collect a broad range of personal and sensitive data, including emotional states, therapy history, behavioral patterns, and even biometric data. However, the regulatory gaps in South Africa’s third-party data-sharing laws have led to several high-profile privacy breaches. A particularly alarming case is the National Health Laboratory Service (NHLS) data breach in South Africa, where sensitive health records of thousands of patients were leaked due to insufficient cybersecurity measures and unauthorized third-party access (Cassim and Chapanduka, 2024). This incident highlights the urgent need for stricter oversight into data-sharing agreements, particularly when personal health information is involved.

In addition to large-scale breaches, there have been numerous global cases where mental health apps have mishandled user data. For example, investigations into popular AI-driven therapy apps such as BetterHelp and Talkspace have revealed that user conversations and mental health assessments were shared with third-party advertisers without clear consent (Thandayuthapani, 2025). These cases underscore how weak third-party data governance allows for the commodification of sensitive mental health information, often without the user’s knowledge. In South Africa, where digital literacy varies and regulatory enforcement is inconsistent, similar risks persist. Without stronger legislative safeguards, mental health app users remain vulnerable to exploitation, reinforcing the need for mandatory transparency reports, stronger security protocols, and explicit user controls over data-sharing practices.

## 4 Regulatory landscape in South Africa

While South Africa’s regulatory framework, particularly the Protection of Personal Information Act (POPIA), National Health Act (NHA), and the Consumer Protection Act (CPA), provides a solid foundation for protecting personal and health data, there are significant gaps in addressing the specific legal and ethical challenges associated with mental health applications. These include the need for more nuanced regulation of mental health data, third-party data sharing, ethical considerations in digital mental health, and protections for emerging technologies like neurotechnology.

POPIA mandates that personal information, including health data, must be processed lawfully and reasonably, without infringing on privacy (Section 4) (South African Government, 2013). For mental health apps, explicit, voluntary, informed, and specific consent must be obtained before collecting sensitive health data, as required by Section 11. Section 10 emphasizes data minimization, meaning that only data essential for providing mental health services should be collected to prevent over-collection and misuse. Users also have rights to access, correct, or delete their data under Sections 23–25. Organizations are required to implement security measures to protect personal data from loss, damage, unauthorized access, or unlawful processing (Section 19), which is critical given the sensitivity of mental health data. However, mental health data is not explicitly distinguished from other health data in POPIA, despite its additional sensitivities related to psychological conditions and emotional health. There is also limited guidance on anonymization or pseudonymization when sharing such data with third parties, including for research or analytics purposes. POPIA does not sufficiently regulate third-party data sharing, including relationships with cloud providers or analytics companies, which may handle data for app improvement or marketing purposes. Additionally, POPIA lacks clarity on the regulation of AI-driven decision-making systems used by mental health apps, raising concerns about profiling and biased decisions. Cross-border data transfers pose another challenge, as POPIA restricts such transfers to countries with similar protections but does not offer guidance on risks associated with storing mental health data in jurisdictions lacking equivalent laws. Compliance across multiple countries with differing data privacy laws remains an issue. While POPIA mandates security measures, it does not provide detailed standards for digital mental health platforms, leaving vulnerabilities in mobile devices, cloud services, or transmitted data. Furthermore, POPIA does not specify how mental health app providers should handle breaches, especially concerning sensitive data. Finally, the right to request data deletion is complicated by the need to retain mental health records for ongoing treatment, and POPIA does not clarify when deletion requests may be denied.

The NHA regulates the provision of healthcare services and includes important provisions for the protection of patients’ health information, including data collected through digital platforms (South African Government, 2003). It mandates that healthcare providers, including those offering services via mental health apps, maintain the confidentiality of patient information, ensuring that only authorized individuals have access to this data (Section 14). Additionally, the Act requires explicit patient consent or legal authorization before any health records, including mental health data, can be shared with third parties (Section 15). Healthcare practitioners are also responsible for taking measures to prevent unauthorized access to health records, meaning that mental health apps must be implemented strong security mechanisms to protect sensitive data (Section 17). However, the NHA has some gaps, particularly regarding digital platforms. It does not provide specific guidelines on how mental health apps should safeguard data, particularly with respect to third-party access, cloud storage, or international data transfers. The Act also lacks guidance related to the use of AI or automated tools for collecting mental health data. Moreover, the NHA does not address the complexities of obtaining informed consent from users of mental health apps, especially when mental health conditions may affect their ability to fully understand consent agreements.

The CPA (South African Government, 2008) provides protections for consumers’ personal information, including data collected by businesses offering mental health services via apps. Section 69 of the CPA emphasizes consumer privacy, requiring businesses to obtain consent before collecting or disclosing personal information. Mental health app providers must take reasonable steps to prevent unauthorized access to this data. The Act also states that businesses ensure the accuracy and fair use of the personal information they collect, such as mental health assessments, and that this data is used only for its intended purposes (Sections 22–24). Additionally, Section 41 requires suppliers, including mental health app providers, to be transparent about how they use consumers’ data. Businesses must provide clear and understandable privacy policies, particularly when dealing with sensitive mental health information. However, the current regulatory framework does not go far enough in requiring mental health application providers to clearly disclose their third-party data-sharing practices. This is particularly concerning given the highly sensitive nature of mental health data, which, if shared with external entities such as advertisers, analytics firms, or third-party service providers, could lead to potential privacy violations, discrimination, or commercial exploitation. At present, many mental health apps bundle their data-sharing agreements within general terms of service, making it difficult for users to understand exactly who has access to their personal data and how it is being used.

To address these gaps, several amendments should be introduced to the CPA to strengthen data transparency, user control, and regulatory enforcement mechanisms. Firstly, mandatory third-party data disclosure statements should be required for all mental health applications. These statements should explicitly list all third parties with whom user data is shared and describe the specific purpose of data processing. To ensure consistency and accessibility, the South African government should develop a standardized data-sharing disclosure template that all firms must use. Additionally, companies should be obligated to submit periodic reports to the Information Regulator, detailing their third-party data-sharing practices, the types of data exchanged, and any changes made to their privacy policies. Secondly, the current consent mechanisms under the CPA should be enhanced to provide stronger protection for mental health data. A two-tiered consent framework should be introduced, requiring separate and explicit opt-in consent for sharing mental health data, rather than allowing it to be bundled with general data-sharing agreements. Users should also be given the ability to revoke their consent at any time, with a clear and easily accessible option to modify their data-sharing preferences within the app settings. This would ensure that individuals retain ongoing control over their mental health information.

Another critical area that requires reform is the monetization of mental health data. Currently, the CPA does not prevent mental health applications from profiting from users’ data, potentially allowing sensitive information to be sold to third parties for commercial gain. A new provision should explicitly prohibit the sale or monetization of mental health data without direct and informed user compensation. If firms engage in data monetization practices, they should be required to provide detailed transparency reports explaining how the data is anonymized and the specific purposes for which it is used.

Enforcement mechanisms under the CPA should also be significantly strengthened to deter non-compliance and ensure accountability. Financial penalties for companies that fail to disclose third-party data-sharing practices should be increased, taking inspiration from the GDPR, where fines can be calculated as a percentage of global annual revenue. Additionally, a dedicated regulatory oversight unit within the National Consumer Commission (NCC) should be established to conduct compliance audits on digital mental health applications. This unit should be tasked with investigating violations, enforcing penalties, and ensuring that mental health data is handled in accordance with the law. To further protect users, the CPA should introduce a requirement for mental health application providers to conduct regular Data Protection Impact Assessments (DPIAs). These assessments should identify potential privacy risks associated with data collection, storage, and sharing, and should outline mitigation strategies to ensure compliance with legal and ethical standards. Companies should be mandated to submit these assessments to the Information Regulator on an annual basis, providing ongoing oversight of mental health data protection practices.

To implement these reforms effectively, South African legislators should take several key steps. First, the CPA should be amended to include a dedicated section on digital mental health data protection, ensuring that existing privacy protections are adapted to the specific risks posed by mental health applications. Second, policymakers should work to harmonize the CPA with the POPIA and the NHA to avoid regulatory inconsistencies and provide a clear, unified legal framework for digital mental health data governance. Additionally, a public register of mental health application providers operating in South Africa should be created, allowing users to verify which platforms comply with data protection regulations. To further address the risks posed by AI-driven mental health tools, the CPA should introduce legal requirements for AI transparency and ethical auditing, ensuring that any algorithm-based mental health assessments or recommendations are based on evidence-based standards and do not lead to biased or harmful outcomes.

The Medicines and Related Substances Act (South African Government, 1965) primarily focuses on regulating the safety, efficacy, and quality of medicines and medical devices. Its applicability to mental health applications is limited. The Act applies to digital tools and apps that are classified as medical devices, particularly those providing diagnostic tools, monitoring health conditions, or offering treatment, such as AI-driven mental health interventions. These apps would need to comply with the regulatory framework for medical devices, including licensing and safety standards. However, many mental health apps that offer general wellness advice, mood tracking, or meditation do not fall under the strict definition of medical devices. As a result, they are not covered by the Act. Apps that provide information related to medicines, such as managing psychiatric medications or offering advice on drug use could be subject to the Act’s regulations, particularly regarding the accuracy of the information provided. There are several regulatory gaps in the Act. First, it does not regulate mental health apps focused on general well-being or emotional support, leaving many such apps unregulated. Second, there is ambiguity in classifying certain mental health apps, particularly those using AI for mental health advice, creating uncertainty around compliance and user safety. Furthermore, the Act does not provide guidance for the use of AI-driven tools or automated decision-making in mental health apps, raising concerns about profiling and potential bias. The lack of clear regulations in these areas mean that many mental health apps, especially those not classified as medical devices are left without comprehensive oversight.

As AI-driven technologies increasingly shape mental health interventions, ensuring transparency, accountability, and fairness in automated decision-making is critical. Many mental health applications rely on AI to analyze user behavior, assess emotional states, and even provide therapeutic recommendations. However, these systems are often black-box models, meaning that their decision-making processes are not easily interpretable, which raises concerns about accuracy, bias, and accountability. To prevent algorithmic bias and ensure ethical AI deployment, South African regulators should introduce mandatory AI transparency requirements. Mental health app providers should be required to disclose how their AI models are trained, what data they process, and whether they are subject to human oversight. Additionally, regular external audits of AI-driven mental health applications should be mandated to identify potential biases in decision-making, particularly in racially culturally, and linguistically diverse populations. Further, an independent AI Ethics Review Board should be established to evaluate the fairness, safety, and effectiveness of mental health algorithms before they are widely deployed. These regulatory measures will help build public trust in AI-driven mental health tools, ensuring that automated interventions remain accurate, unbiased, and aligned with ethical standards.

## 5 Discussion and future directions

Digital mental health technologies have the potential to expand mental healthcare access in South Africa, but the current regulatory framework does not sufficiently address critical issues related to privacy, security, consent, and equitable access. In particular, there is a significant gap in how sensitive mental health data is managed in multi-party agreements, leading to increased risks of data misuse. Additionally, disadvantaged populations, including those with low digital literacy, face barriers in understanding complex consent mechanisms, making them more vulnerable to privacy violations. To ensure that digital mental health solutions are both effective and inclusive, policymakers must adopt clearer regulatory guidelines, culturally sensitive approaches, and community-driven solutions.

One of the key areas requiring reform is simplifying informed consent mechanisms. Many mental health applications currently use long and complex terms of service agreements, which are difficult for users, especially those with limited digital literacy, to understand. To address this, layered consent interfaces should be introduced, where key information is presented in clear, plain language before detailed terms are provided. Additionally, consent processes should be available in all major South African languages to enhance accessibility. Granular consent options should also be mandated, allowing users to approve specific types of data collection rather than giving blanket consent. Furthermore, users should have the ability to easily modify or revoke consent at any time, ensuring they retain full control over their mental health data.

Beyond consent, culturally sensitive design and community participation are essential to ensuring that digital mental health applications reach a diverse population. Many South African communities remain hesitant to engage with digital mental health tools due to concerns about privacy, stigma, and trust. To address this, mental health applications should be developed with direct input from local communities to ensure that they align with user needs, cultural expectations, and linguistic preferences. Additionally, the integration of anonymous and offline access options can make these platforms more accessible to users who may fear social stigma. The government should also invest in digital literacy programs, particularly in rural and underserved areas, to ensure that individuals understand how to use mental health apps safely and effectively.

Another major challenge is ensuring robust data privacy and security in multi-party agreements. While POPIA requires user consent for data sharing, it does not adequately regulate how mental health data should be handled across multiple stakeholders, such as AI developers, cloud service providers, and data analytics firms. To mitigate these risks, South Africa could introduce Standardized Data Protection Agreements (DPAs) that clearly outline data-sharing restrictions, security protocols, and retention policies. AI-driven mental health applications should also be required to disclose how their algorithms function and undergo external audits to prevent bias, errors, or data misuse. Additionally, regulations should strictly limit cross-border data transfers, ensuring that South African users’ mental health data is not stored or processed in jurisdictions with weaker privacy laws.

Finally, to enforce these changes, the CPA and other relevant laws must be strengthened. Regulatory penalties for non-compliance with data privacy laws should be significantly increased, following models such as the GDPR. Additionally, a dedicated regulatory unit within the NCC should be established to audit mental health application providers and ensure compliance with strengthened legal standards. South Africa must modernize its legal framework for digital mental health technologies to enhance data privacy protections, simplify consent mechanisms, and promote culturally inclusive practices. Strengthening multi-party data governance, improving AI transparency, and empowering users with better digital literacy tools will be critical steps in building a more ethical and accessible digital mental health ecosystem. By implementing these targeted legal and policy reforms, South Africa can ensure that mental health data remains protected while fostering responsible innovation in digital healthcare.
